# Residential Radon in Central and South America: A Systematic Review

**DOI:** 10.3390/ijerph17124550

**Published:** 2020-06-24

**Authors:** Alexandra Giraldo-Osorio, Alberto Ruano-Ravina, Leonor Varela-Lema, Juan M. Barros-Dios, Mónica Pérez-Ríos

**Affiliations:** 1 Department of Preventive Medicine and Public Health, University of Santiago de Compostela, 15782 Santiago de Compostela, Spain; alexandra.giraldo.osorio@usc.es (A.G.-O.); juanm.barros@usc.es (J.M.B.-D.); monica.perez.rios@usc.es (M.P.-R.); 2 Grupo de Investigación en Promoción de la Salud y Prevención de Enfermedades, Departamento de Salud Pública, Universidad de Caldas, Manizales 170002, Colombia; 3 Consortium for Biomedical Research in Epidemiology & Public Health (*CIBER en Epidemiología and Salud Pública/CIBERESP*), 15782 Santiago de Compostela, Spain; 4 Galician Health Technology Assessment Agency, Health Knowledge Management Agency (*Axencia de Coñecemento en Saúde/ACIS*), Galician Regional Health Authority, 15706 Santiago de Compostela, Spain; avalia-t1@sergas.es

**Keywords:** radon, lung neoplasms, lung cancer risk in never-smokers, residential radon, natural radiation

## Abstract

Radon gas is a pulmonary carcinogen and the second leading cause of lung cancer after smoking. There are many countries that have not implemented measures to reduce the risk it poses to the general population. The aim of this study was to locate available evidence on exposure to residential radon and the regulations to monitor and control this across Central and South America, by conducting a review of the scientific literature and government documents in accordance with the Preferred Reporting Items for Systematic Reviews and Meta-Analyses (PRISMA) guidelines. This review included 31 studies which had taken measurements of radon in these countries. While Brazil, Argentina, and Peru have undertaken most research, no country in Central and South America has a national map of exposure to residential radon. The prevalence of exposure to radon was uneven, both among the different countries and within individual countries. No country has regulations to prevent the entry of radon into homes, and nine countries have not set maximum permissible concentrations for residential radon. There is a limited number of studies in South and Central America, with a limited spatial coverage, and there is a need to improve knowledge on exposure to residential radon and its effects, and for governments to take the necessary actions to introduce preventive measures in their statutory regulations.

## 1. Introduction

Radon is a gas that originates from the disintegration of uranium contained in rocks forming part of the Earth’s crust. It is colorless, odorless, and tasteless, and in the process of decay emits an alpha form of ionizing radiation. Radon has a number of isotopes, the most relevant of which from an epidemiologic standpoint is ^222^Rn, whose parent element in the radioactive decay chain is uranium 238. ^222^Rn has a mean life of 3.8 days and is transformed into short half-life daughters that also emit alpha radiation, i.e., 218polonium and 214polonium [1]. Homes constructed in places where the rocks in the Earth’s crust contain high uranium concentrations have a greater likelihood of experiencing high indoor radon concentrations, if they are not well insulated from the subsoil [2]. Indoor radon can be measured using different devices and technologies, and the most used and reliable to determine residential radon concentration is based on counting alpha particles impacting on a surface (alpha-track devices or Solid State Nuclear Track Devices) [3]. These detectors have to be placed for a minimum period of 3 months.

Numerous studies have associated occupational and residential exposure to radon gas with a higher risk of developing lung cancer, with 6 to 18% of all cases being attributed to this cause [4,5,6,7,8]. As a risk factor for lung cancer, radon is ranked second after smoking and first among never-smokers [2,7,9], and is recognized by the International Agency for Research on Cancer (IARC) as a carcinogenic agent in humans [10]. Radon maps have been plotted in Europe and North America, which not only make it possible to identify geographic areas with higher radon concentrations, but also serve to define risk areas [11] and establish different levels of prevention measures. In many countries, such prevention measures are contained in legislation that addresses this public health problem [12].

In Central and South America, lung cancer is one of the most frequent cancers and the leading cause of cancer-related death in both sexes [13,14]. Across the period 1985–2008, lung cancer mortality was the leading or second leading cause of death among men in half of the countries in Central America and 80% of the countries in South America [13]. In women, the pattern was different: the countries reporting the highest lung cancer mortality rates were Venezuela, Argentina, Colombia, and Brazil [13]. 

Given the epidemiologic relevance of lung cancer in Central and South America, and the important role played by interior radon in the disease’s appearance, it is crucial to ascertain what information there is on exposure to residential radon and what regulations have been established to govern radon in these countries. This information will make it possible to strengthen or initiate prevention activities aimed at reducing exposure to this carcinogen in a geographic area inhabited by more than 400 million people.

The main objective of the present study is to review the scientific evidence available on studies performed in Central and South America describing residential radon exposure, and also to describe regulations and by-laws enacted on preventive measures and maximum allowable indoor radon concentrations in such countries. 

## 2. Materials and Methods 

To carry out this study, we undertook a systematic review of the literature in accordance with the Preferred Reporting Items for Systematic Reviews and Meta-Analyses (PRISMA) guidelines [15]. 

### 2.1. Search Strategy

A bibliographic search was conducted in the PubMed (MedLine), Embase, Latin American & Caribbean Health Sciences Literature (LILACS), and Biblioteca Virtual en Salud (BVS) databases and via the Google Scholar search engine, using search strategies that combined MeSH terms and free-text terms as follows: ((“radon”[MeSH Terms] OR “radon”[All Fields]) AND (“South America”[All Fields] OR “south america”[MeSH Terms])), ((“radon”[MeSH Terms] OR “radon”[All Fields]) AND (“Central America”[All Fields] OR “central america”[MeSH Terms])). In addition, further combinations were made with the names of the respective countries of Central America (Guatemala, Honduras, El Salvador, Nicaragua, Costa Rica, Panamá OR Panama) and South America (Colombia, Venezuela, Brasil OR Brazil, Perú OR Peru, Ecuador, Bolivia, Uruguay, Paraguay, Chile, Argentina). A thorough search was also made of government ministry websites hosted by the respective study countries, seeking all relevant documents, protocols, and administrative documents. Where necessary, we contacted the authors of scientific papers and reports to ascertain the existence of additional unpublished research. No time limit was set, and all publications available in the databases until February 2020 were included. We considered papers in international peer-reviewed papers and other types of papers, such as conference papers, papers in national journals, or documentation uploaded in institutional websites. The citations contained in the papers published were reviewed, and no language limits were imposed. Special emphasis was laid on the comprehensiveness of the search, so as to be sure to include all relevant information, albeit at the risk of obtaining irrelevant data that would subsequently have to be discarded.

### 2.2. Study Selection and Eligibility Criteria

The initial selection of papers was made on the basis of the information contained in the title or abstract shown on the register. To qualify for inclusion, studies were required to report a minimum of 10 measurements of residential radon in a rural or urban setting in one or more Central and South American countries or some geographic area of these. Studies that included more than 3 provinces or districts were treated as nation-wide studies. There were no restrictions in terms of type of study or publication. In the case of any paper that successfully passed the first filter, its full text was requested for the purpose of verifying its compliance with the eligibility criteria. 

When it came to statutory rules and regulations, only government documents were considered, with the exclusion of all publications by scientific societies, groups of experts, regions, counties, or any other entity at a subnational level. Similarly, regulations governing occupational exposure to radon were not included, since this aspect fell outside the scope of our review. We also excluded, because it was outside the scope of this research, radon presence in kindergartens, schools, public places, hospitals, or other non-residential settings.

### 2.3. Data-Extraction, Management and Data Synthesis

The same information was extracted from all the papers included, using a data-extraction table for the purpose. Data were obtained on the following variables: country; method of measurement; geographic area (province, municipality, district); number of provinces, municipalities, or districts; setting (urban, rural); number of homes measured; and radon concentration in becquerels per cubic meter (Bq/m^3^) (maximum, minimum and average, geometric mean or median). Information was extracted from each reference by two team members, with any disagreements or discrepancies in interpretation of the data being settled by consensus. A qualitative data synthesis was performed. 

## 3. Results

### 3.1. Summary of Studies Included

**Figure 1:**
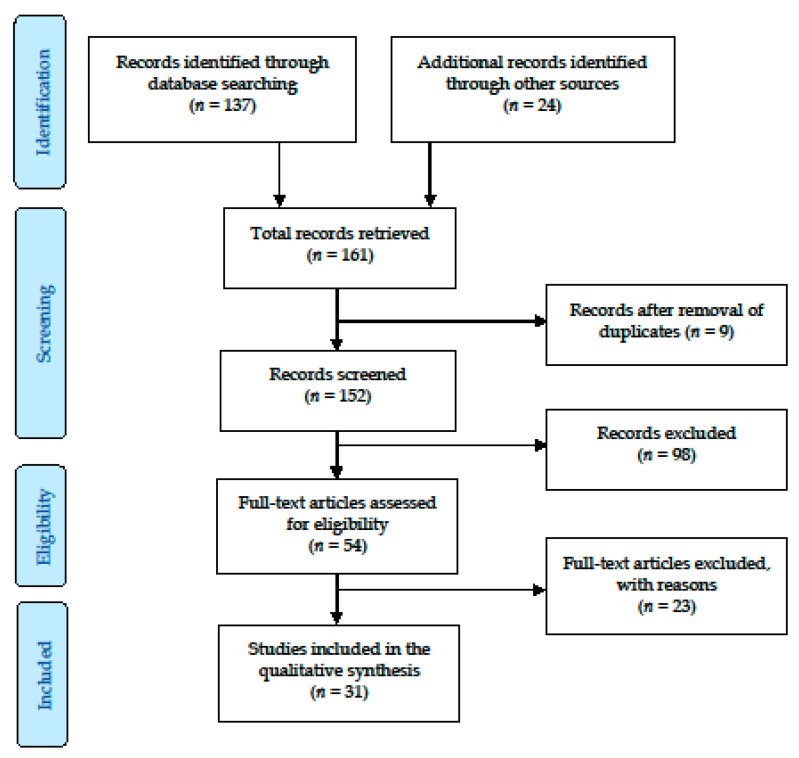
Flowchart of the included studies describing indoor radon concentrations.

Figure 1 shows the flowchart of the published papers describing indoor radon concentration retrieved. A total of 31 studies fulfilled the inclusion criteria. Most of the documents located were papers on scientific reviews. The first study to target residential radon dated from 1982 and was carried out in Rio de Janeiro, Brazil [16]. The greater part of the records corresponded to measurements of residential radon exposure made in Brazil, with 15 studies, and in Argentina and Peru, with six studies in each. 

### 3.2. Information Available on Residential Exposure to Radon 

Table 1 gives a description of the studies selected, broken down by year of publication. In the Central American region, residential radon measurements were solely found for Costa Rica [17], while in South America they were found for Argentina [18,19,20,21,22,23], Brazil [16,21,23,24,25,26,27,28,29,30,31,32,33,34,35], Chile [20,36], Colombia [37], Ecuador [21,23,38,39], Paraguay [20], Peru [21,23,40,41,42,43], and Venezuela [21,44]. Most of the studies involved radon measurements taken in areas or regions within a given country. There was wide variation in the type of area (district, municipality, province, etc.) and setting (urban, rural, or both) where measurements were made and in the number of measurements taken. The number of homes measured by the studies identified, ranged from 13 through 2689. The highest number of measurements corresponded to Argentina: until 1998, 1630 measurements had been taken, with this figure rising to 2689 by 2006 [19,21,22,23]. Only three studies measured radon in more than 300 homes [16,21,22]. 

The majority of measurements were made using the alpha-track technique, and essentially corresponded to studies of urban settings. Residential radon levels ranged from 0 Bq/m^3^ through 3723 Bq/m^3^. The highest levels were found in Brazil, and specifically in the following areas: Lages Pintadas, 3723 Bq/m^3^ [32] and 2893 Bq/m^3^ [33]; Belo Horizonte 2671 Bq/m^3^ [31]; and Poços de Caldas, 1024 Bq/m^3^ [26]. Brazil was also the country in which individual studies generally observed higher radon concentrations. 

Radon concentrations found for each of the countries were highly variable, with Costa Rica showing the lowest concentrations and Brazil the highest. Nevertheless, since some of the included studies were performed in radon-prone areas, these results are not valid to produce a national average. 

### 3.3. Government Directives or Plans Governing Residential Radon Exposure and its Regulation

Table 2 shows information on the regulations in force in each country. Three South American countries stipulated maximum reference levels in their legislation (Bolivia, Colombia, and Peru). In two Central American countries (Guatemala and Nicaragua) and two South American countries (Paraguay and Uruguay), residential radon action levels were in line with international guidelines: in the case of Guatemala, Nicaragua, and Paraguay, these were in accordance with the International Atomic Energy Agency (IAEA) Safety Series No. 115; and in the case of Uruguay, these were in accordance with the International Commission on Radiological Protection (ICRP) 1993 report No 65. In another nine countries, there was no mention of such radon reference levels for homes. 

Differences were also observed between the action levels of countries that had established statutory levels. Hence, Bolivia and Colombia had a limit of up to 400 Bq/m^3^, which was the most restrictive level of all countries across Central and South America, whereas Guatemala, Nicaragua, Paraguay, Peru, and Uruguay had the highest action level, i.e., up to 600 Bq/m^3^. All of the Central and South America countries had statutory regulations governing ionizing radiations, but without specific allusion to the maximum level of exposure permitted. No legal rules or regulations were detected covering the prevention of accumulation of residential radon in any of the countries analyzed. 

## 4. Discussion

To our knowledge, this is the first systematic review to address the presence of residential radon in homes across Central and South America. It highlights the fact that very few measurements have been made in these countries and, very important, that the action level in all those countries which have set a statutory threshold is above 300 Bq/m^3^, the maximum recommended by the World Health Organization (WHO) [47]. According to the studies reviewed, residential radon concentrations vary widely among the different countries and even within individual countries. These results also highlight the lack of legislation or statutory provisions targeted at preventing or blocking the seepage of radon into homes across Central and South America.

The studies reviewed report widely differing results on residential radon exposure. Thus, the high interior radon levels in the town of Lages Pintadas (Brazil) are linked to the fact that the town lies on a number of outcrops of pegmatite ore bodies, which are naturally enriched with uranium [33]. This agrees with what is already known about the way radon levels vary globally [48] according to bedrock characteristics [49]. It is noteworthy that the mean values recorded for Brazil are very close to those registered in Mexico, with 140 Bq/m^3^ [47].

Our review located no data published on residential radon concentrations in any country in Central America, except Costa Rica. This is in line with the United Nations Scientific Committee on the Effects of Atomic Radiation (UNSCEAR) report (published in 2009), which provides evidence of residential radon action levels for countries on all continents, except Central America. It does however cite action levels for some South American countries, sourced from the studies by Canoba et al. (2002) and Zeeb (2007), with Canoba’s being the most representative study to have been conducted in the region, owing to its inclusion of six countries and the number of measurements made [45,50].

Brazil is the country in which most measurements have been made to assess exposure to residential radon. This may be due to the fact that from 1990 through 1994 it was the regional coordinating country for Latin America within the framework of the “Radon in the Human Environment” research program [51]. It should be borne in mind here that Brazil is the most populated and the largest country in Central and South America.

The greater use of the track detector method for measurement of residential radon can be explained by the fact that track detectors are passive devices requiring neither electric current nor a pump to function in the sampling environment, whereas active devices require electricity and enable continuous monitoring and recording of radon gas concentrations and fluctuations across the measurement period [47]. In addition, track detectors are cheaper than other types of detectors, and extremely reliable if used with the appropriate methodology [52] Many of the studies reviewed did not report the geometric mean or median, both of which are important measures, since radon concentrations tend to follow a log-normal distribution. Six studies were excluded after a reading of the full text because they measured radon in fewer than 10 homes. These studies were performed in countries already having indoor radon studies included in this review, and all of them had convenience sampling.

We did not find relevant differences between urban and rural settings. Though this can be seen as striking at first sight, some of the settings considered as urban included dwellings with low stories (i.e., ground floor and first floor), making them not very different to rural settings. Furthermore, some of the settings considered urban were located in areas with moderate radon concentrations exhaling from the soil, therefore somewhat high radon concentrations were expected.

Unlike countries in Europe, which have legislation governing recommended action levels, of the 16 countries that make up Central and South America, only seven have regulations that specifically indicate residential radon levels. Even so, in countries such as Guatemala, Nicaragua, Paraguay, Peru, and Uruguay, the maximum levels are up to 600 Bq/m3, a figure equivalent to double that recommended in Europe [53], four times that recommended in the USA [54], and six times the ideal level suggested by the WHO [47].

The above results are not in line with the indications and recommendations issued by international institutions such as the ICRP, the European Atomic Energy Community (EURATOM), and UNSCEAR, to the effect that it falls to the state to issue regulations, take measurements, and make assessments in respect of ionizing and other radiation [50,55].

Apart from legislation stipulating residential radon action levels, it is also important to have laws governing home construction in order to protect people from radon, since high ^222^Rn concentrations in homes can be reduced by means of corrective measures. These measures are aimed at preventing ^222^Rn from seeping into homes/buildings from the soil, or eliminating it through better ventilation of interior spaces. No such legislation was, however, found for any of the Central and South American countries.

Notwithstanding the rigor of the search, the great diversity of websites from which the legislation-related documents were drawn (whether for sourcing recommended radon levels or building ordinances) makes it impossible to rule out the likelihood of regulations existing in any of the countries for which no statutory provisions were found. Moreover, it is always possible that such rules and regulations might be in the drafting phase.

It is important to highlight that the available evidence, besides scarce, is extremely heterogeneous in the number of radon measurements per study, methodology of measurement, and settings considered (rural, urban, radon-prone areas). This fact underlines the need to have nationwide studies allowing the robust description of indoor radon concentrations within a certain country. A further limitation is related to the period of measurement, with some studies having very short radon integration time compared to others, and a lack of full consideration of seasonal radon variations, a relevant issue in certain settings [56].

## 5. Conclusions

In general, it can be concluded that none of the countries analyzed has a global public health action targeted at tackling the problem of residential radon. The fact that only three studies have measured radon in more than 300 homes and the limited number of studies available means that in all of Central and South America there are very few initiatives of this type. As evidence of this, it should be noted that none of the countries in Central and South America has a national map of residential radon exposure or a unified legislative approach to combat the presence of radon in homes. There is also a very limited special coverage of residential indoor mapping, and WHO recommendations regarding indoor radon have not been enforced in any legislation of the analyzed countries. Part of the explanation for this may lie in the fact that, to date, these countries have had other health priorities, but now the time has come to address the problem of residential radon, given its association with lung cancer and the known interaction it displays with the smoking habit. It is vital that public administrations, health professionals, and the general public be made aware of the problem of residential radon, so that the necessary measures can be implemented to reduce its effects on health.

## Figures and Tables

**Table 1:** Description of radon measurement studies in Central and South American countries.

Authors (Publication)	Measurement Year	Method of Detection	Location *	Coverage	No. of Areas **	Setting	No. of Homes	Radon Level (Bq/m^3^)				
								Max	Min	AM	GM	MED
Urban M, et al. (1985) [16]	1982	Alpha-track	Brazil	Nationwide	4	Urban	320	67	24	114	-	-
Gomez J C, et al. (1990) [18,20]	1983–1985, 1987–1988	Alpha-track	Argentina, Buenos Aires	Autonomous city	1	Urban	102	-	-	23	-	-
Gomez J C, et al. (1990) [18,20]	1983–1985	Alpha-track	Argentina, San Rafael	Province	1	Urban and rural	53	-	-	50.5	-	-
Gomez J C, et al. (1990) [18,20]	1984–1985	Alpha-track	Argentina, Malargüe	Province	1	Urban and rural	24	-	-	34	-	-
Loría LG, et al. (1993) [17]	-	Alpha-track	Costa Rica	Nationwide	25	Urban	45	30.3	8.1	13.85	-	-
Stuardo E (1996) [20,36]	1992–1993	Alpha-track, electrets	Chile, Santiago	Municipality	1	Urban	119	86	4	24.5	-	-
Malanca A, et al. (1997) [34]	1995–1996	Alpha-track	Brazil, Natal	Municipality	1	Urban	24	40	3.2	15.4	11.7	-
Malanca A, et al. (1997) [35]	1995–1996	Alpha-track	Brazil, Rio Grande del Norte and Ceará	State	2	Urban	88	140	3.2	11.9	12.4	11.9
De Paula Melo, V (1999) [24]	1997–1998	Alpha-track	Brazil, Monte Alegre	Municipality	1	Urban	33	188	27	75	-	-
De Paula Melo, V (1999) [24]	1997–1998	Alpha-track	Brazil, Inglés de Souza	Village	1	Rural	35	200	32	116	-	-
Sajó-Bohus L, et al. (1999) [21,44,45,46]	1993	Alpha-track, gamma radiation	Venezuela	Nationwide	1	Urban	36	41	31	36	-	-
Guedes S, et al. (1999) [25]	1999	Alpha-track	Brazil, Campinas	Municipality	1	Urban	15	150.9	6.2	43.98	-	-
UNSCEAR (2000) [20]	-	-	Paraguay	-	-	-	51	-	-	28	-	-
Canoba A, et al. (2001) [21,45]	2000	Alpha-track	Brazil, Campinas and Presidente Prudente	Municipality	2	-	320	275.73	16.68	81.27	-	-
Canoba A, et al. (2001) [21,45,46]	2000	Electrets	Ecuador, Quito	Municipality	1	-	61	187.78	35.9	94.3	-	-
Canoba A, et al. (2001) [21,45,46]	2000	Alpha-track	Peru, Lima	Municipality	1	-	168	46.43	22.14	64.59	-	-
Salazar S (2002) [37]	2002	Electrets	Colombia, Manizales	Municipality	1	Urban and rural	18	11.1	166.5	67.71	-	-
Magalhães M, et al. (2003) [27]	1996–1997	Alpha-track	Brazil, Rio de Janeiro	Municipality	1	Urban	48	200	5	-	40	-
Magalhães M, et al. (2003) [27]	1996–1997	Alpha-track	Brazil, Poços de Caldas	Municipality	1	Urban and rural	125	985	31	-	132.5	-
Veiga LH, et al. (2003) [26]	2000	Alpha-track	Brazil, Poços de Caldas	Municipality	1	Urban and rural	138	1024	12	96	140.5	-
Paulo SR, et al. (2005) [28]	2002	Alpha-track	Brazil, Poços de Caldas	Municipality	1	Urban	39	193	2	133	117	-
Canoba AC, et al. (2006) [19,21,22,23,45,46]	1983–2006	Alpha-track, activated carbon, electrets	Argentina	Nationwide	14	-	2689	300	-	41.6	-	-
De Olveira Santos T, et al. (2007) [29]	-	Continuous monitoring, electrets	Brazil, Belo Horizonte	Municipality	1	Urban	13	306	12.5	77.49	-	-
Zeeb H (2007) [23]	2005	Alpha-track, electrets	Ecuador	Nationwide	1	-	-	400	100	-	-	-
Zeeb H (2007) [23]	2005	Alpha-track	Peru	Nationwide	1	-	-	600	200	-	-	-
Hadler J, et al. (2008) [30]	1996–1997	Alpha-track	Brazil, Campinas	Municipality	1	Urban	70	286	11.8	80.6	-	-
Santos TO, et al. (2009) [31]	-	Continuous monitoring, electrets	Brazil, Belo Horizonte	Municipality	1	Urban	13	2671.4	18.5	148	128.2	-
Thomas Campos, et al. (2011) [32]	-	Electrets, gamma radiation	Brazil, Lages Pintadas	Municipality	1	Urban and rural	100	3723	20	376	358	-
Campos TFC, et al. (2013) [33]	-	Electrets	Brazil, Lages Pintadas	Municipality	1	Urban rural	210	2893	15	566	291	288
Pereyra P et al. (2015) [40,41]	2014	Alpha-track	Peru, Lima	Province	1	Urban	97	598.25	9.5	183	-	-
Cuadrado C, Carrasco J (2016) [39]	2016	Electronic detector	Ecuador, Riobamba	Municipality	1	Urban	14	95	2	32	-	-
Liza Neciosup RA (2017) [42]	2015–2016	Alpha-track	Peru - Lima, San Martín de Porres	District	1	Urban	125	218.9	103	155.6	154.1	155.1
Vega Cabrera BO (2017) [43]	2015–2017	Alpha-track	Peru-Lima, San Luis	District	1	Urban	84	124	44	68	56	-
Loayza Cabrera MJ (2018) [38]	2017–2018	Alpha-track	Ecuador, Cuenca	Municipality	1	Urban	47	201.11	1.11	35	-	-

* Location: indicates the country of South America (Peru, Ecuador, Venezuela, Brazil, Argentina, Chile, Paraguay, Colombia) or Central America (Costa Rica). ** Areas: Political administrative division or jurisdiction in which the measurements were made (i.e., district, municipality, province, etc.). AM: arithmetic mean; GM: geometric mean; MED: median; –: no data. UNSCEAR United Nations Scientific Committee on the Effects of Atomic Radiation

**Table 2:** Governmental regulations, ordinances, or plans governing residential radon exposure and home construction.

Country	Regulation	Year of Publication	Body that Lays down the Regulation	Action Level for Chronic Exposure to Radon in Homes	Building Regulations for Control of Radon Levels
Bolivia (South America)	Supreme Decree 19172: Radiological Protection and Safety Act	1982	Bolivian Institute of Science and Nuclear Technology: the national authority with competence in matters relating to the use of ionizing radiations	Annual concentration of 400 Bq/m^3^	Where radon concentrations in home interiors exceed 400 Bq/m^3^, engineering solutions must be adopted for ventilating living spaces and reducing emanations of the gas.
Colombia (South America)	Resolution 18–1434: Radiological Protection and Safety Regulations	2002	Ministry of Mines and Energy	Mean annual concentration of 400 Bq/m^3^	-
Guatemala (Central America)	Government Resolution No. 55-2001: Radiological Protection and Safety Regulations pursuant to the Control, Use and Application of Radioisotopes and Ionizing Radiations Act	2001	Ministry of Mines and Energy President of the Republic	Optimized action levels are, in general, in line with the guidelines issued by the International Atomic Energy Agency (IAEA), i.e., 200–600 Bq/m^3^ (assuming 7000 h per year in interiors and an equilibrium factor of 0.4.)	Management, taking into account the legal and social circumstances applicable, must decide on the compulsoriness of corrective actions for situations of chronic exposure in homes.
Nicaragua (Central America)	Technical Directive No. 001-2011: technical directive, technical regulations for protection against ionizing radiations	2011	National Atomic Energy Commission	It establishes radon limits according to the International Atomic Energy Agency (IAEA) Safety Series No. 115, namely: 200–600 Bq/m^3^ (assuming 7000 h per year in interiors and an equilibrium factor of 0.4.)	In situations of chronic exposure in homes, the regulatory authority or intervening body must decide whether the remedial actions are of a mandatory nature or only in the form of a recommendation, taking into account the existing social and legal context.
Paraguay (South America)	National safety regulations for protection against ionizing radiations and safety of radiation sources	2001	Ministry of Public Health and Social Welfare. National Atomic Energy Commission	It establishes radon limits according to the International Atomic Energy Agency (IAEA) Safety Series No. 115, namely: 200–600 Bq/m^3^ (assuming 7000 h per year in interiors and an equilibrium factor of 0.4.)	-
Peru (South America)	Supreme Decree No. 009-97-EM: Radiological Safety Regulations	1997	President of the Republic. Peruvian Institute of Nuclear Energy	Mean annual concentration of 200 to 600 Bq/m^3^	-
Uruguay (South America)	Resolution 016/2014: Regulation UY 100, basic Radiological Protection and Safety Regulations. 8th Revision	2018	National Radiological Protection Authority. Ministry of Industry, Energy and Mining.	It establishes radon limits according to the International Commission on Radiological Protection (ICRP) 1993 report No 65, namely: 200–600 Bq/m^3^ (assuming 7000 h per year in interiors and an equilibrium factor of 0.4.)	
